# Supplementary material for: Potential Nosocomial Infections by the Zika and Chikungunya Viruses in Public Health Facilities in the Metropolitan Area of Recife, Brazil
Source: Trop Med Infect Dis. 2022 Nov 4;7(11):351. doi: 10.3390/tropicalmed7110351 (PMC9694620; doi:10.3390/tropicalmed7110351)
Supplement: Supplementary file 1 [file tropicalmed-07-00351-s001.zip › tropicalmed-1885433-supplementary.pdf]

Tropical Medicine and Infectious Disease

MDPI

Article

Potential Nosocomial Infections by the Zika and Chikungunya Viruses in Public Health Facilities in the Metropolitan Area of Recife, Brazil

Larissa Krokovsky [1], Duschinka Ribeiro Duarte Guedes [1], Fabiana Cristina Fulco Santos [1], Kamila Gaudêncio da Silva Sales [2], Daniela Anastácio Bandeira [3], Claudenice Ramos Pontes [3], Walter Soares Leal [4], Constância Flávia Junqueira Ayres [1] and Marcelo Henrique Santos Paiva [1,5,*]

[1] Entomology Department, Aggeu Magalhães Institute, Oswaldo Cruz Foundation, Recife, 50740-465, PE, Brazil
[2] Immunology Department, Aggeu Magalhães Institute, Oswaldo Cruz Foundation Recife, 50740-465, PE, Brazil
[3] Pernambuco's Health Department, Recife, 50751-530, PE, Brazil
[4] Department of Molecular and Cellular Biology, University of California, Davis, CA 95616, USA
[5] Life Sciences Center, Agreste Academic Center, Federal University of Pernambuco, Caruaru, 55002-970, PE, Brazil
* Correspondence: marcelo.paiva@fiocruz.br; Tel.: +55-81-21012552

Table S1. Primers and probes sequences used in DENV, CHIKV and ZIKV detection.

Virus	Dye	Oligonucleotide Name	Sequence (5′–3′)	Amplicon Size (bp)	Reference
DENV	CY5	DENVG-F	AAGGACTAGAGGTTAGAGGAGACCC	108	[1]
		DENVG-R	CGTTCTGTGCCTGGAATGATG		
		DENVG-P	CY5-AACAGCATATTGACGCTGGGAGAGACCAGA-MGB		
ZIKV	FAM	ZIKV 1163c	CCACTAACGTTCTTTTGCAGACAT	77	[2]
		ZIKV 1087	CCGCTGCCCAACACAAG		
		ZIKV 1108 P	FAM-AGCCTACCTTGACAAGCAGTCAGACACTCAA-MGB		
CHIKV	VIC	CHIKV 6856	TCACTCCCTGTTGGACTTGATAGA	126	[3]
		CHIKV 6981	TTGACGAGAGTTAGGAACATACC		
		CHIKV 6919 P	VIC-AGGTACGCGCTTCAAGTTCGGCG-BHQ1		

Table S2. Linear regression analysis values of standard curves for DENV, ZIKV and CHIKV in singleplex and multiplex assays.

Virus	Parameters	RT-qPCR Assays	
		Singleplex	Multiplex
DENV	Slope	−3.608	−3.362
	R^2	0.997366667	0.998
	E%	89.398	98.369
	Y-inter	60.88	59.899
ZIKV	Slope	−3.536	−3.406
	R^2	0.997	0.996
	E%	91.799	96.610
	Y-inter	62.75	62.30
CHIKV	Slope	−3.497799953	−3.448
	R^2	0.994066676	0.996
	E%	93.2	95.118
	Y-inter	61.50	62.589

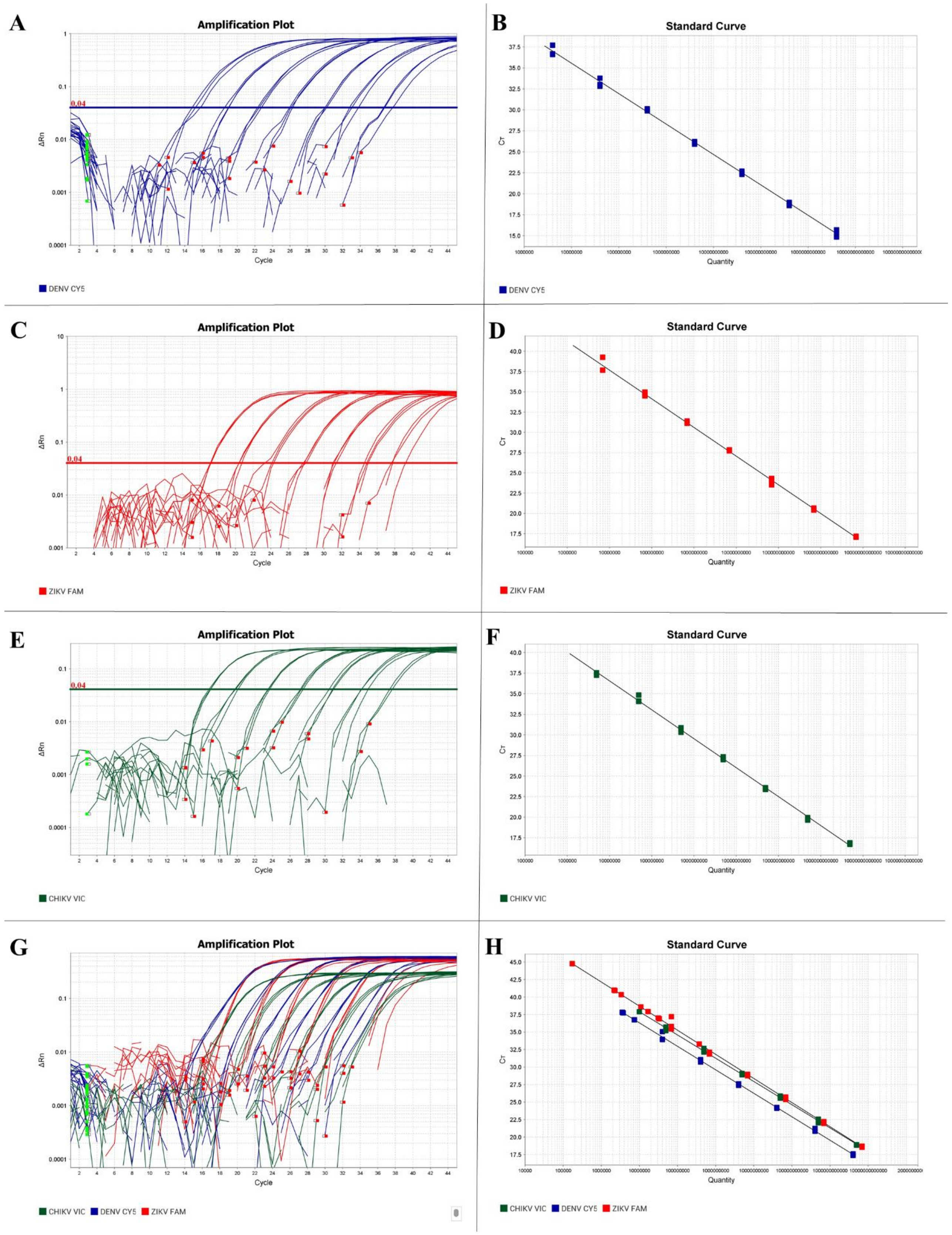

Figure S1. Amplification plot and standard curve of the singleplex and multiplex real-time RT-PCR assays. **A–B:** Singleplex for DENV; **C–D:** Singleplex for ZIKV; **E–F:** Singleplex for CHIKV; **G–H:** Multiplex for DENV, ZIKV and CHIKV. .

References

1. Magalhaes, T.; Braga, C.; Cordeiro, M.T.; Oliveira, A.L.S.; Castanha, P.M.S.; Maciel, A.P.R.; Amancio, N.M.L.; Gouveia, P.N.; Peixoto-da-Silva, V.J.; Peixoto, T.F.L.; et al. Zika Virus Displacement by a Chikungunya Outbreak in Recife, Brazil. *PLoS Negl Trop Dis* **2017**, *11*, e0006055, doi:10.1371/journal.pntd.0006055.
2. Lanciotti, R.S.; Kosoy, O.L.; Laven, J.J.; Velez, J.O.; Lambert, A.J.; Johnson, A.J.; Stanfield, S.M.; Duffy, M.R. Genetic and Serologic Properties of Zika Virus Associated with an Epidemic, Yap State, Micronesia, 2007. *Emerg Infect Dis* **2008**, *14*, 1232–1239, doi:10.3201/eid1408.080287.
3. Lanciotti, R.S.; Kosoy, O.L.; Laven, J.J.; Panella, A.J.; Velez, J.O.; Lambert, A.J.; Campbell, G.L. Chikungunya Virus in US Travelers Returning from India, 2006. *Emerg Infect Dis* **2007**, *13*, 764–767, doi:10.3201/eid1305.070015.
